# Intelligent Tourism Information Service Model considering Tourist Experience in the Environment of Internet of Things

**DOI:** 10.1155/2022/5252218

**Published:** 2022-04-28

**Authors:** Han Liu

**Affiliations:** ^1^School of Education and Management, Bozhou Vocational and Technical College, Bozhou 236800, Anhui, China; ^2^Gujing School of Management, Bozhou 236800, Anhui, China

## Abstract

Due to the narrow range of route search and the single choice of the optimal route during the peak period of tourists' tourism, resulting in a long walking time and relatively high route cost, an intelligent tourism information service model considering tourists' experience is proposed in the environment of Internet of things. Firstly, the overall architecture of the Internet of things environment is designed to obtain the characteristic data of the carrying capacity of scenic spots, including tourism resources, psychology, ecology, and economic carrying capacity, based on which the tourist perception experience model is constructed; different service functions such as in-depth mining of Web content, personalized information push, and data information conversion under the environment of Internet of things and the construction of intelligent tourism information service model are realized. The experimental results show that the passenger flow prediction error of the designed model is small and has a good prediction effect of tourist attraction selection demand.

## 1. Introduction

Driven by the rapid development of information technology and Internet technology, tourism has ushered in a golden period of rapid growth. With the gradual development of the national economy and the increase in leisure time and disposable income, tourism has become an important way of entertainment for residents [1]. Because ecotourism has the characteristics of objectivity, dynamics, and management, it is difficult to measure its environmental carrying capacity. There are still great disputes on the selection of indicators and influencing factors [2]. In the open Internet of things environment, the total amount of data information shows a geometric growth, which causes great resistance to the rapid retrieval service of data information [3]. In the Internet of things environment, it is impossible to quickly confirm the current location and return to the specified node. Secondly, due to the limited receiving and holding capacity of information users, it is difficult to correctly understand and apply information [4,5]. Although the emergence of various search engines has satisfied the basic needs of information users to a certain extent, the quality of information retrieved is poor due to the huge amount of Internet information and the existence of information overload [6, 7]. Considering the tourist experience, the intelligent tourism information service model is established, which not only has the function of fast retrieval and collection but also integrates the Internet of things retrieval functions such as personalized database, favorites, and hyperlinks, which significantly improves the information retrieval efficiency.

Reference [8] presents an assessment of the comparative advantage shown by Ukraine's export of tourism services to the EU. For those countries where tourism is an important source of national income and a job-creating activity, the question of tourism and the revitalization of the tourism sector become urgent following the devastating consequences of the pandemic. Tourism stimulates the development of small- and medium-sized enterprises, has great potential for a creative economy, enables rapid recovery of costs, has a significant environmental impact, and enables a high degree of social inclusion, including the use of women and youth labour. Reference [9] proposed the impact of applied quality standards on improving environmental tourism services in nature reserves in southern Jordan. The study population included tourists from all nature reserves in southern Jordan. Descriptive analysis was used to conduct a field survey of 600 visitors to a nature reserve in southern Jordan, selected by a simple random method, through questionnaires. Using statistical analysis of social science statistical procedure according to the results, in the south of Jordan nature reserves, tourist service quality standards to improve the environment have a significant statistical effect, and the study suggested southern Jordan nature reserve management department to make a plan, through continuous application of quality standards, to ensure to improve its ecological tourism services.

Although the above research has made some progress, there is less consideration of tourists' experience. Therefore, an intelligent tourism information service model considering tourists' experience in the Internet of things environment is proposed to adapt. The overall architecture of the Internet of things environment was designed to obtain the characteristic data of tourist attractions' carrying capacity, and based on this, the tourist perception experience model was constructed. Different service functions such as in-depth mining of Web content, personalized information push, and data information conversion can be realized in the Internet of things environment, to adapt and meet the personalized information retrieval and use needs of tourists in the Internet of things environment.

## 2. Intelligent Tourism Information Service Model considering Tourist Experience in the Environment of Internet of Things

### 2.1. Overall Architecture of Internet of Things Environment

The overall architecture of the Internet of things environment can be divided into four main levels of “cloud management edge end.”

#### 2.1.1. Cloud Level

The “cloud” layer can realize the functions of intelligent tourism information service terminal connection, system deployment, data decoupling, and so on, which meets the conditions of high-speed response to the demand for tourism information service and intensive system operation and maintenance [10].

#### 2.1.2. Pipe Level

This layer uses the system of the integration of remote communication network and local communication network to meet the needs of intelligent tourism information service through channel IP, network protocol, and resource self-description.

#### 2.1.3. Edge Level

The architecture of “unified hardware system + edge operation + business software” is used to integrate the functions of network, computing, and storage, combined with edge computing to improve the real-time processing performance and reduce the computing pressure; the terminal is defined according to the software to realize the flexible deployment of intelligent tourism information service [11].

#### 2.1.4. End Level

**Figure 1:**
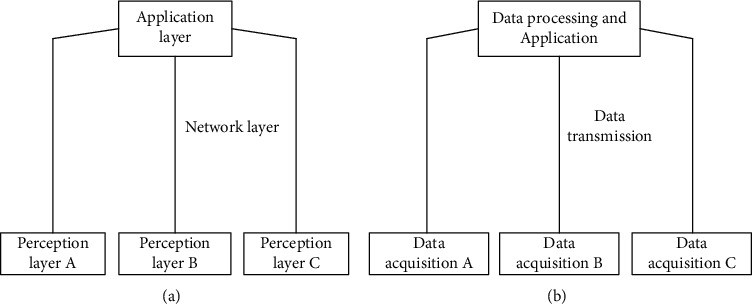
Overall architecture of Internet of things environment.

This level can collect information such as the operation of intelligent tourism information service, equipment, and environmental status and can execute decision-making commands to realize timely communication with tourists. The overall architecture of the Internet of things environment is shown in Figure 1.

### 2.2. Obtain the Characteristic Data of Carrying Capacity of Scenic Spots

From the perspective of tourists' experience and keeping the tourism demand and tourism supply of the scenic spot in a relatively ideal state, the transportation carrying tools of tourists and the facilities or space occupied by them in a specific time are determined through the combination of quantitative and qualitative methods, to obtain the characteristic data of the carrying capacity of the scenic spot, including the carrying capacity data of tourism resources, tourism psychological carrying capacity data, tourism ecological carrying capacity data, and tourism economic carrying capacity data [12,13]. The specific contents of data acquisition are shown in Table 1.

For the data of tourism resource carrying capacity, the calculation formula is as follows:

(1) $$\begin{array}{c} R_{j}=\frac{T_{a}}{T_{1}}\times \frac{S_{a}}{S_{1}}. \end{array}$$

In formula (1), *R*_*j*_ represents the actual limit daily capacity of resources, *T*_*a*_ represents the opening time of tourist destination every day, *T*_1_ represents the actual visiting time of each tourist, *S*_*a*_ represents the resource area, and *S*_1_ represents the minimum space standard of each tourist.

For the data of tourism psychological carrying capacity, the calculation formula is as follows:

(2) $$\begin{array}{c} C_{zl}=\frac{T_{a}}{T_{1}}\times k\times S_{a}. \end{array}$$

In formula (2), *C*_*zl*_ represents daily psychological capacity and *k* represents reasonable capacity per unit area of space.

For the data of tourism ecological carrying capacity, the calculation formula is as follows:

(3) $$\begin{array}{c} S_{tc}=\frac{\sum_{i=1}^{n}S_{i}T_{i}+\sum_{i=1}^{n}Q_{i}}{\sum_{i=1}^{n}P_{i}}. \end{array}$$

In formula (3), *S*_*tc*_ represents the daily ecological capacity, *n* represents the specific number of pollutants, and *S*_*i*_ represents the specific number of *i* pollutants absorbed and purified by the ecological environment, *T*_*i*_ represents the specific purification time of each pollutant, *i* represents the amount of *Q*_*i*_ pollutants treated manually every day, and *i* represents the actual amount of pollutants (type *i*) produced by each tourist every day.

For the data of tourism economic carrying capacity, the calculation formula is as follows:

(4) $$\begin{array}{c} \left\{\begin{array}{c} T_{e}=\frac{\sum_{i-1}^{m}D_{l}}{\sum_{i-1}^{m}E_{l}}\\ T_{h}=\sum_{i-1}^{n\prime }B_{j} \end{array}\right). \end{array}$$

In formula (4), *T*_*e*_ represents the daily carrying capacity of food supply tourism, *m* represents the specific types of food consumed by tourists, *D*_*i*_ represents the supply of *l* kinds of food per day, *E*_*l*_ represents the *l* food demand per person per day, *T*_*h*_ represents the daily carrying capacity of accommodation bed tourism, *B*_*j*_ represents the specific number of accommodation beds in class *j* tourism areas, and *n*′ represents the actual types of accommodation facilities.

### 2.3. Tourist Perception Experience Model

Tourism activities involve many factors, not only taking tourists as the main participation but also taking the image of the scenic spot as the main behavior. Scenic spots attract tourists with specific resources and carry out relevant activities. Whether tourism resources have a certain tourism attraction depends not only on the tourism resources of the scenic spot but also on the comprehensive object environment of the scenic spot [14, 15]. The tourism environment is an important carrier of tourism activities. The tourism environment is one of the important factors for tourists to consider whether the scenic spot is worth traveling. For example, the quality of the natural environment often affects the experience of tourists in this process. If the scenic spot has good tourism resources, but it rains or earthquakes frequently all year round, then tourists will not come to visit. It can be seen that tourism environmental factors are very important.

Tourists bring economic benefits, promote employment, promote the protection of ecological environment and traditional culture, and inevitably bring many negative effects caused by their own lifestyle and behavioral habits to the scenic spot. Therefore, it is of great practical significance to study tourism information services from the perspective of tourists [16].

**Figure 2:**
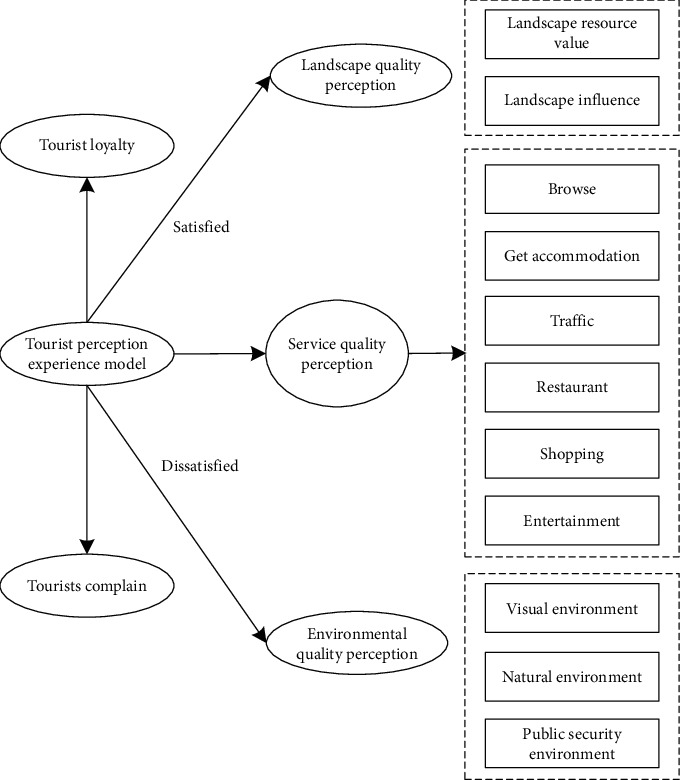
Tourist perception experience model.

ACSI represents the core concept and architecture of tourism motivation, purchase policy, tourism, and tourism process when tourists play. As a dynamic and continuous experience process, the end of tourism can establish a tourist perception experience model through the dynamic process of tourists' comprehensive experience [17, 18]. The specific content is shown in Figure 2.

As can be seen from Figure 2, the tourist perception experience model is built on the basis of tourist resettlement experience. Tourist perception experience is a kind of social perception that tourists perceive the impact of tourism, including the perception of economy, social culture, environment, self, interpersonal relationship, and social role. Tourism experience is generated in every link of tourism product consumption, from landscape appreciation to the acquisition of various services, and the quality of service in each link will affect the quality of tourist experience [19,20]. The accuracy, safety, comfort, and reliability of information transmission determine the service quality of each element. Therefore, tourists can obtain more accurate comprehensive perception experience through the perception of tourism landscape quality, service quality, and environmental quality.

## 3. Realize the Design of Intelligent Tourism Information Service Model considering Tourist Experience

### 3.1. Design of Information Service Function Module

**Figure 3:**
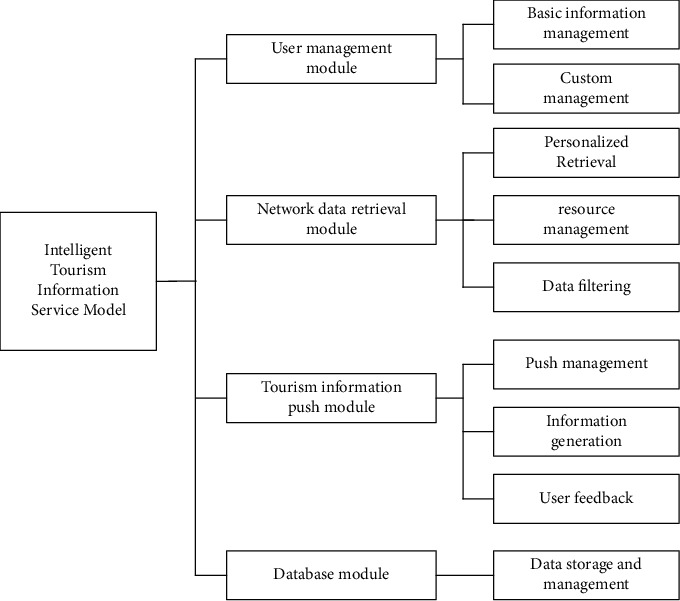
Structure diagram of main functional modules of intelligent tourism information service model.

Consider intelligent tourism information service model of tourist experience, which is divided into four modules, respectively, as tourism information module, the network data retrieval module, push module, and database module, to realize the Internet environment, the depth of the Web content mining, data conversion and other personalized information push service function, the structure of the model, and the main function module design, as shown in Figure 3.

1. *Visitor Module*. The intelligent tourism information service model based on multimode takes tourists as the service center. The functions of the tourist module include basic information management and customized management of tourists' information needs. All personal information and account information of tourists are stored in the module. After authorization, you can enter the account and password to log in to the system. After entering the system, tourists can add, edit, and modify tourist information by themselves after being authorized by the system administrator; through this module, tourists can put forward specific tourism information retrieval needs, customize the database list, and select the retrieval and classification query methods of tourism information. The module has the function of automatic storage and retrieval, and the historical retrieval content can be queried through keywords.
2. *Network Data Retrieval Module*. This module is the core module of the tourism information service model, through which tourists can retrieve and customize the content of interested weblogs [21]. To adapt to the compound multi-data transmission mode, a data retrieval network is designed. The network has good compatibility and supports different modes such as static data transmission and dynamic data transmission. The bus matrix can process 64 bit data input and output in parallel, with four I/O interfaces. The network data retrieval module can deeply mine and analyze the contents of the network log and actively switch the mode of tourism information retrieval according to the use habits and preferences of tourists, to obtain the retrieval habits of tourists and store keywords and characteristic words in the database, to facilitate the subsequent data call of tourists.
3. *Tourism Information Push Module*. The tourism information push module includes the specific functions of pushing tourism information management, tourism information generation, and tourists' use feedback. The total amount of tourism information in the network is huge, and the total amount of data information collected according to tourists' habits and preferences is huge. Push management is to preprocess and classify this redundant and wrong tourism information first, retain useful tourism information, and eliminate interference information [22,23]; through the classified retrieval of customized tourism information for tourists, the most economical tourism information transmission mode is selected in the Internet of things environment, the tourism information categories required by tourists are generated, and directional and regular push is realized [24]. The tourism information push module has a two-way information interaction function; that is, on the one hand, it transmits basic tourism information to tourists, and on the other hand, it also receives tourists' feedback, to optimize each module of the system and continuously improve the database [25].
4. *Database Module*. According to the above three modules, the list category of the database is divided into tourist basic tourism information data, tourist search tourism information table, and personalized data table, as shown in Tables 2–4, respectively.

Other forms in the database are extended and expanded on the basis of the basic form and connected with the basic form through the primary key field, which is convenient for tourists' retrieval and query.

### 3.2. Realize Intelligent Tourism Information Service Model

The Internet of things is taken as the goal of tourists' experience perception, an intelligent tourism information service model is established through intelligent perception, a large number of tourism-related real-time information are mined through the Internet of things, such as scenic spot tickets, accommodation places, weather, and meteorology, this information is gathered and analyzed, and the tourism information available for service is summarized [26,27].

To establish an intelligent tourism information service model, we need to take the big data of the Internet of things as the goal and use multi-agent technology to divide the establishment of the model into multiple modules, namely data acquisition, data perception, data decomposition, data storage, data integration, central control, and data display modules. Among them, data acquisition is responsible for the input of the model, collecting the tourism big data of the Internet of things through the compiled crawler program, making preliminary selection and filtering, and submitting the data to the data decomposition module after completing the preliminary processing; the data decomposition module decomposes the collected data into multiple parts through extraction, filtering, decomposition, and other operations and submits them to the perception module [28]; after sensing the tourism information, the perception module in the platform selects the tourism data with service value in a unified format through weighted calculation and normalization processing and then integrates this data information with service value into independent data through the data integration module, which is stored in the data storage module, managed storage, and provided to the data display module, and the data are displayed in front of tourists.

The central control module is responsible for connecting each module in the perception model, controlling the information exchange of each module, and the processed request link queue established by the manager in the process of obtaining the tourism data of the Internet of things, so that the whole platform can effectively process the tourism big data and communicate information [29].

In the above, the data collection is mainly realized through the Web page analysis algorithm, which is not simply judged according to whether all the text of the Web page is related to the given subject. This judgment method lacks certain reliability. In the process of establishing the intelligent tourism information service model [30], the data collection is mainly based on the theme relevance of the parent Web page of the Internet of things. The correlation between the current Web page and the theme and the location of the Web page link is comprehensively determined. The final result may be a continuous value between 0 and 1. The closer the value is, the higher the correlation between the collected data and tourism service information, which proves that the collected data are the target data of the platform [31,32].

Assuming that the 0/1 mode is used to represent whether tourists have been to the scenic spot, the check-in times of tourists correspond to the degree of interest of tourists in the scenic spot. Therefore, the similarity between tourist interest points is solved using the check-in times of tourists in the scenic spot, and the expression is as follows:

(5) $$\begin{array}{c} X_{qd}=\frac{\left(\sum y_{i,j}\times y_{k,j}\right)}{\sum y_{i,j}}. \end{array}$$

In formula (5), *y*_*i*,*j*_ represents the check-in frequency of a tourist *y*_*i*,*j*_ in scenic spot *j* and *y*_*k*,*j*_ represents the check-in frequency of another tourist in scenic spot *j*.

The first *N*′ tourists with the greatest similarity are selected according to the similarity between tourists. These tourists are used to form a set *U*′, and the possibility that tourists will go to the scenic spot without checking in is solved according to the collaborative filtering model based on tourists. Then, the formula is as follows:

(6) $$\begin{array}{c} Y_{qd}=\frac{\left(\sum X_{qd}\times y_{k,j}\right)}{\sum X_{q\;d}}. \end{array}$$

Assuming that a tourist has been to *m* cities, the expression of the number of interest points that can be mined is obtained according to the sign-in times and stay time of his activity track:

(7) $$\begin{array}{c} Z_{qd}=\left(\frac{\sum l_{c}}{m}\right)\times X_{qd}\times Y_{qd}. \end{array}$$

In formula (7), *l*_*c*_ represents the vector set represented by the points of interest. To ensure that the mining of interest points is comprehensive enough, it is necessary to use the characteristics of tourist similarity, social relations, and geographical location information to realize the mining [33,34]. Because the probability values of interest points are different, the standardized weighting method should be used for processing during integration, and the processing results should be substituted into the model of mining interest points. The construction of intelligent tourism information service model can be realized [35,36], and its expression is as follows:

(8) $$\begin{array}{c} S_{ab}=P_{ab}\times Z_{qd}\times \lambda . \end{array}$$

In formula (8), *P*_*ab*_ represents the collection of users' check-in interest points, and *λ* represents the training parameters. Intelligent tourism is a highly complex and dynamic field. Intelligent tourism information service providers often need to rely on models to predict the marketing objectives of the tourism market and explore the potential market. Users also need tourism information to assist decision-making. These need to be realized through the organization and integration of intelligent tourism information service models. There is an urgent need for an Internet of things processing technology to organize them in depth. In the Internet of things environment, various network information technologies are used as a structured data application specification, to realize the construction of intelligent tourism information service model considering tourist experience in the Internet of things environment.

## 4. Experimental Analysis

**Figure 4:**
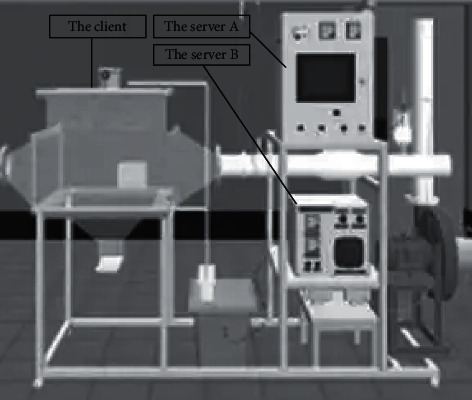
Environment diagram of simulation experimental platform.

In the experimental platform of this study, the CPU is Intel (*R*) Q4800, the frequency is 2.66 GHz, the computer memory is 512 GB, the simulation programming environment is MATLAB 2016 under windows 10 system, the classifier uses LIBSVM and grid search method for parameter optimization, and the kernel function used is Gaussian kernel. The experimental equipment includes two servers and one client, which stores the communication transmission information in the cloud. The experimental environment is shown in Figure 4.

**Figure 5:**
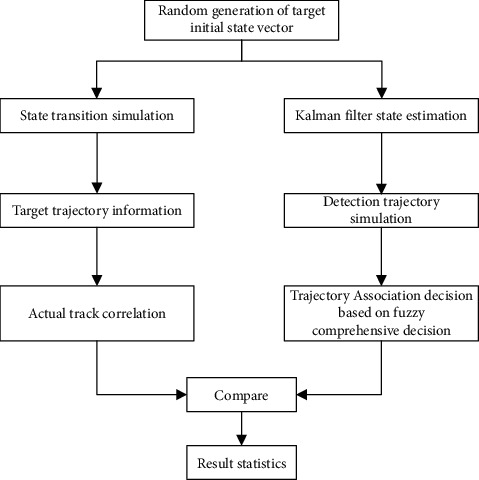
Simulation principle of intelligent tourism information service model.

According to Figure 4, to ensure the time synchronization between sampling and updating, the time can be calibrated by smoothing and extrapolation. To ensure the reliability of the experiment, the experiment is carried out according to the method in Figure 5.

In the simulation platform, the initial parameters of the sensor are set: the position coordinate is (*X*, *Y*)=(120*km*, 120*km*), the observation radius *r*_1_ is 100 km, the ranging error *σ*_*l*_1__ is 150m, and the angle measurement error *σ*_*θ*_1__ is 1°. In the simulation experiment, five data sets of wine, forest, glass, iris, and segmentation used for identification in the UCI standard database are used. The basic information of the data set is shown in Table 5.

Under the five authoritative data sets in Table 5 above, the model in this study, the model in reference [8], and the model in reference [9] are used for experimental verification, respectively. The experimental environmental parameters are shown in Table 6.

**Figure 6:**
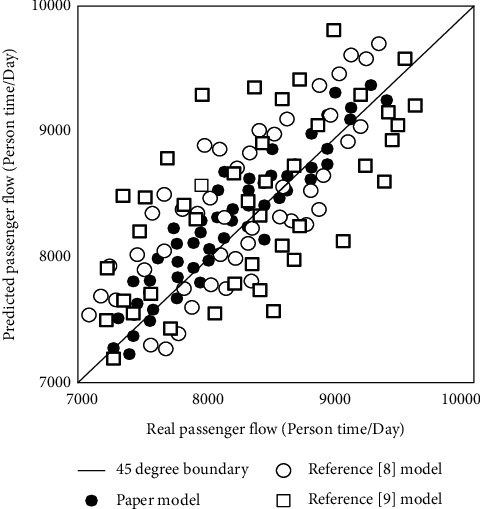
Scatter diagram of model prediction period.

The dynamic virtual variable of the intelligent tourism information service model is set to 0, and the three models are used to predict the passenger flow of a scenic spot on a working day and compared with the actual value. The overall scatter diagram of the predicted passenger flow and the real passenger flow is shown in Figure 6.

According to the analysis of Figure 6, the scattered points of the passenger flow prediction results of the model in this study are roughly distributed at the 45° boundary, while the scattered points of the passenger flow prediction results of the model in reference [8] and the model in reference [9] are relatively scattered. This shows that the passenger flow prediction error of this model is small and has good effect.

To test the intelligent tourism information service performance of the model in this study, 2021 is taken as an example to predict the demand for tourists for scenic spot selection. The prediction results are shown in Table 7.

**Figure 7:**
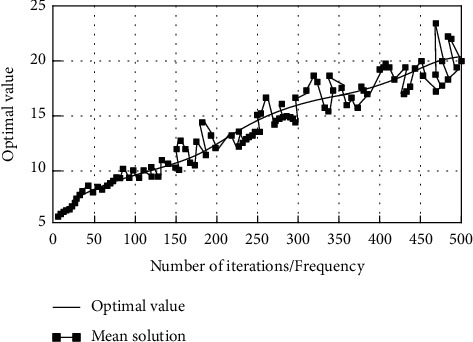
Optimal travel route search results.

As shown in Table 2, the relative error between the number of tourists choosing scenic spots and the actual number of tourists in 2021 predicted by the model in this study is less than 6%. The experimental results show that the model in this study has a good prediction effect on the demand for selecting scenic spots. The method in this study is used to search the optimal tourism route, and the results are shown in Figure 7.

According to Figure 7, with the increase in iteration algebra, the optimal solution of the search results of the optimal tourism route shows an upward trend, the average value fluctuates up and down in the optimal solution, and the fluctuation range of iteration oscillation is small. When the number of iterations reaches 470 generations, the peak value of iteration fluctuation appears, indicating that the optimal path has been found and has good convergence.

To sum up, the prediction error of the model in this study is small and has a good prediction effect. The intelligent tourism information service model considering tourist experience in the Internet of things environment can effectively find the optimal path.

## 5. Conclusions and Prospects

### 5.1. Conclusions

1. Under the environment of Internet of things, the prediction error of passenger flow of intelligent tourism information service model considering tourist experience is small
2. The intelligent tourism information service model has a good effect on the demand prediction of tourist attraction selection
3. When the number of iterations reaches 470 generations, the peak value of iteration fluctuation appears, indicating that the intelligent tourism information service model considering tourist experience in the designed Internet of things environment has found the optimal path

### 5.2. Prospects

1. As the infrastructure construction of intelligent tourism information service is still relatively backward, which is the biggest obstacle to the intelligent development of tourism industry, in the future, we can design intelligent tourism information service system under the background of intelligent tourism and use all kinds of network information technology under the environment of Internet of things to provide intelligent tourism services for tourists. Therefore, whether the establishment of the model can be truly implemented remains to be verified by the development of the times.
2. The next step is to establish an intelligent tourism information service model based on the tourism consumption model, user needs, and functional needs, through the current situation and investigation and analysis, and establish an intelligent tourism information service model considering the experience of tourists using the service design methods such as tourist experience map, tourist role model, and interview. At the same time, it also needs to be verified by testing.
3. To facilitate the travel needs of tourists, the operation mode of intelligent tourism information service model is set as tourism app, the main functional modules of tourism app are determined according to the priority of tourist behavior path and service contact point, the corresponding service blueprint and app information architecture diagram are drawn, and the interactive interface prototype of tourism app is designed in combination with the design principles of interactive interface, and the visual design is improved.

## Figures and Tables

**Table 1:** Specific contents of data acquisition.

Serial number	Data type	Specific content of data
1	Tourism resources carrying capacity	Actual limit daily capacity of resources
2	Tourism psychological carrying capacity	When tourists can get greater satisfaction, the maximum tourism activity capacity that the region can carry
3	Tourism ecological carrying capacity	The ability to treat tourism pollutants through artificial methods and ecological environment absorption and purification methods
4	Tourism economic carrying capacity	It refers to the reception capacity of basic tourism facilities, related facilities, and industrial supporting facilities, represented by the actual supply capacity of entertainment and accommodation facilities

**Table 2:** Basic tourism information data of tourists.

Field	Meaning	Type	Length	Can it be blank	Primary key
UID	Number	Int	16	No	Yes
Name	Full name	Var	10	No	No
Sex	Gender	Var	4	No	No
Bd	Birth	Date	10	No	No
Add	Date	Var	50	Yes	No
Edu	Address	Var	40	Yes	No

**Table 3:** Tourist information retrieval.

Field	Meaning	Type	Length	Can it be blank	Primary key
UID	Number	Int	16	No	Yes
Id	Data	Int	20	No	Yes
Cta	Number	Int	20	No	No
Ctn	Classification	Var	10	No	No
Cat	Number	Var	8	Yes	No

**Table 4:** Personalized datasheet.

Field	Meaning	Type	Length	Can it be blank	Primary key
UID	Number	Int	16	No	Yes
Id	Data	Int	20	No	Yes
Kw	Number	Int	20	Yes	No
Ty	Key tourism information number	Var	20	Yes	No
Ind	Data	Flo	10	Yes	No

**Table 5:** Basic information of data set.

Data set	Sample input dimension	Number of training samples	Number of test samples	Number of identifications
Wine	24	133	133	4
Forest	47	247	317	7
Glass	18	159	114	2
Iris	11	121	84	3
Segmentation	37	331	3310	5

**Table 6:** Experimental environmental parameters.

Experimental configuration	Experimental parameters
CPU	Dual-core 2 GHz
Effective memory, memory	3.4 GB, 4 GB
The server	ASUA RS100-X5

**Table 7:** Forecast results of tourists' demand for scenic spot selection.

Scenic spot category	Actual person times	Predicted person times	Absolute quantity difference	Relative difference (%)
Resort	10222	11433	1221	5.6
Ecological farm	25340	24200	1140	2.3
Water hole	34586	35442	−856	‐1.2
Drift	38351	37607	744	0.9
Picking point	21004	19412	1592	3.9

## Data Availability

The raw data supporting the conclusions of this article will be made available by the authors, without undue reservation.
